# Hepatitis B Virus X Protein Is Stabilized by the Deubiquitinating Enzyme VCPIP1 in a Ubiquitin-Independent Manner by Recruiting the 26S Proteasome Subunit PSMC3

**DOI:** 10.1128/jvi.00611-22

**Published:** 2022-06-13

**Authors:** Qiong Wu, Lu Zhang, Xiazhen Xu, Yi Zhang, Jiajian Shi, Xu Lin, Wannan Chen

**Affiliations:** a Key Laboratory of Gastrointestinal Cancer (Fujian Medical University), Ministry of Education, School of Basic Medical Sciences, Fujian Medical University, Fuzhou, China; b Fujian Key Laboratory of Tumor Microbiology, Department of Medical Microbiology, School of Basic Medical Sciences, Fujian Medical University, Fuzhou, China; University of Southern California

**Keywords:** hepatitis B virus, HBx protein, VCPIP1, PSMC3, ubiquitin-independent proteasome pathway

## Abstract

Hepatitis B virus (HBV)-related hepatocellular carcinoma (HCC) is the sixth most common cancer worldwide, and the viral X protein (HBx) is an etiological factor in HCC development. HBx is a high-turnover protein, but knowledge of the role of deubiquitinating enzymes (DUBs) in maintaining HBx homeostasis is very limited. We used a 74-DUB library-based yeast two-hybrid assay and determined that a novel DUB, valosin-containing protein-interacting protein 1 (VCPIP1), interacted with HBx. VCPIP1 and its C-terminal amino acids 863 to 1221 upregulated the HBx protein expression, with or without HBV infection. Mechanistically, VCPIP1 stabilized HBx protein through a ubiquitin-independent pathway, which was validated by the HBx ubiquitination site mutant plasmid. Coimmunoprecipitation assays demonstrated the potency of VCPIP1 in recruiting 26S proteasome regulatory subunit 6A (PSMC3) and forming a ternary complex with HBx through mutual interaction. *In vitro*, purified His-tagged PSMC3 protein rescued HBx degradation induced by the 20S proteasome, and *in vivo* VCPIP1 synergized the mechanism. Functionally, HBx specifically binding to VCPIP1 significantly enhanced the transcriptional transactivation of HBx by activating NF-κB, AP-1, and SP-1 and inhibited hepatoma cell clonogenicity in Huh7 and HepG2 cells. Moreover, we further demonstrated that overexpression of VCPIP1 significantly affected the HBV covalently closed circular DNA (cccDNA) transcription in HBV-infected HepG2-NTCP cells. Altogether, our results indicate a novel mechanism by which VCPIP1 recruits PSMC3 to bind with HBx, stabilizing it in a ubiquitin-independent manner, which might be critical for developing DUB inhibitors in the future.

**IMPORTANCE** HBx is a multifunctional viral oncoprotein that plays an essential role in the viral life cycle and hepatocarcinogenesis. HBx degradation occurs through the ubiquitin-proteasome system (UPS). However, whether novel compartments of the DUBs in the UPS also act in regulating HBx stability is not fully understood. Here, for the first time, we defined VCPIP1 as a novel DUB for preventing HBx degradation by the 20S proteasome in a ubiquitin-independent manner. PSMC3, encoding the 26S proteasome regulatory subunit, directly stabilized HBx through physical binding instead of a common approach in protein degradation, serving as the key downstream effector of VCPIP1 on HBx. Therefore, the ternary binding pattern between VCPIP1, HBx, and PSMC3 is initiated for the first time, which eventually promotes HBx stability and its functions. Our findings provide novel insights into host-virus cross talk by targeting DUBs in the UPS.

## INTRODUCTION

Hepatocellular carcinoma (HCC) is the sixth highest malignant tumor and the fourth leading cause of cancer-related mortality worldwide (1). Chronic hepatitis B virus (HBV) infection accounts etiologically for 80% of HCC cases, especially in East Asia (2). As HBV is the most representative member of the oncovirus family, the understanding of the mechanisms by which HBV is pathogenically involved and contributes to multiple aspects of HBV-related HCC has rapidly advanced in the past decade. Hepatitis B X protein (HBx) encoded by the HBV genome is commonly recognized as an important viral oncoprotein (3). Transgenic HBx exhibits potency for inducing HCC occurrence in mice independently of other factors (4). As a multifaceted transactivator protein, HBx can stimulate the expression of various proto-oncogenes, including c-Fos, c-Myc, and c-Jun, thereby participating in the vital oncogenic processes of promoting hepatocyte proliferation, inhibiting DNA damage responses and apoptosis, and inducing protein degradation and HBV replication (5). Upon HBV infection, the viral genome is delivered into the nucleus and transformed into covalently closed circular DNA (cccDNA), serving as the transcription template for all HBV viral RNAs. cccDNA persistence in the nucleus of infected hepatocytes is critical for HBV chronicity (6). Consistently, numerous studies in the literature agree that HBx is essential for initiating and maintaining transcription of HBV cccDNA (7), but the mechanisms are still poorly understood.

Clinical researches have presented HBx as a possible diagnostic marker by demonstrating its expression in 40% of sera and 85% of liver tissue samples from patients with HCC (8). Therefore, targeting HBx could be a promising therapeutic strategy. HBx hijacking of the ubiquitin-proteasome system (UPS) continues to emerge as a central theme around virus-induced oncogenesis (9). Meanwhile, HBx stability is also influenced by versatile UPS components through direct or indirect mechanisms, highlighting the need for deep understanding of HBV-UPS interaction to develop UPS-targeting treatments (10). HBx is an unstable protein that is polyubiquitinated and subsequently degraded by the proteasome through ubiquitin-dependent and -independent ways (11). Different from the common knowledge of ubiquitinase-induced HBx degradation by the p53-mediated E3 ubiquitin ligase Siah-1 (12), DNA-binding protein inhibitor ID-1 (Id-1) recruitment of proteasome subunit C8 (PSMA3) (13), and the X-linked tumor suppressor (TSPX)-interacting 19S lid subunit RPN3 (14), the emerging role of deubiquitinases (DUBs) in HBx homeostasis remains largely unknown. Generally, DUBs cleave polyubiquitin chains or completely remove them from ubiquitinated proteins and generate recycling-free ubiquitin; therefore, they have important functions in regulating the ubiquitin-dependent pathways (15). We previously demonstrated that a representative DUB, ubiquitin-specific peptidase 15 (USP15), in the ubiquitin-specific protease class, protected HBx from proteasome-mediated degradation in a ubiquitin-dependent manner (16). To date, more than 100 DUBs have been identified, and evidence is accumulating for their functions in viral infectious diseases (17), but their functions still need to be fully described in the field of HBV.

In the present study, we utilized a high-throughput yeast two-hybrid library consisting of 74 DUBs to identify the novel DUBs responsible for HBx stability. Based on the yeast two-hybrid assay, a critical member in the ovarian tumor (*OTU*) protease class of DUBs, valosin-containing protein-interacting protein 1 (VCPIP1), was identified as a potential interacting molecule of HBx. The direct binding between VCPIP1 and HBx was verified by an *in vitro* glutathione *S*-transferase (GST) pulldown assay, and their physical interaction was validated by an *in vivo* coimmunoprecipitation (Co-IP) assay and confocal microscopy assay. Furthermore, amino acid (aa) residues 121 to 154 of HBx and aa residues 863 to 1221 of VCPIP1 were required for the interaction. Functionally, ectopic VCPIP1 expression increased HBx expression in a dose-dependent manner and in the context of HBV infection. The increased HBx resulted from significant stabilization by VCPIP1 overexpression, along with the proteasome inhibitor MG132, thereby prolonging its half-life. More interestingly, we identified a novel role of VCPIP1 in stabilizing HBx in a ubiquitin-independent manner by simultaneously forming a larger complex with HBx and the intracellular free PSMC3, which may ultimately inhibit HBx degradation. Finally, the VCPIP1-induced HBx stability greatly promoted its canonical transcriptional activities and contributed to the inhibition of colony formation of Huh7 and HepG2 cells. We also demonstrated that VCPIP1 overexpression promoted the HBV cccDNA transcription and enhanced the HBV gene expression in the HBV-infected HepG2-NTCP cells. Collectively, detailed deciphering of the interplay between the host UPS and HBx viral oncoprotein may indicate the potential of DUB inhibitors in the future.

## RESULTS

### VCPIP1 is a novel HBx-binding protein via direct interaction.

**FIG 1:**
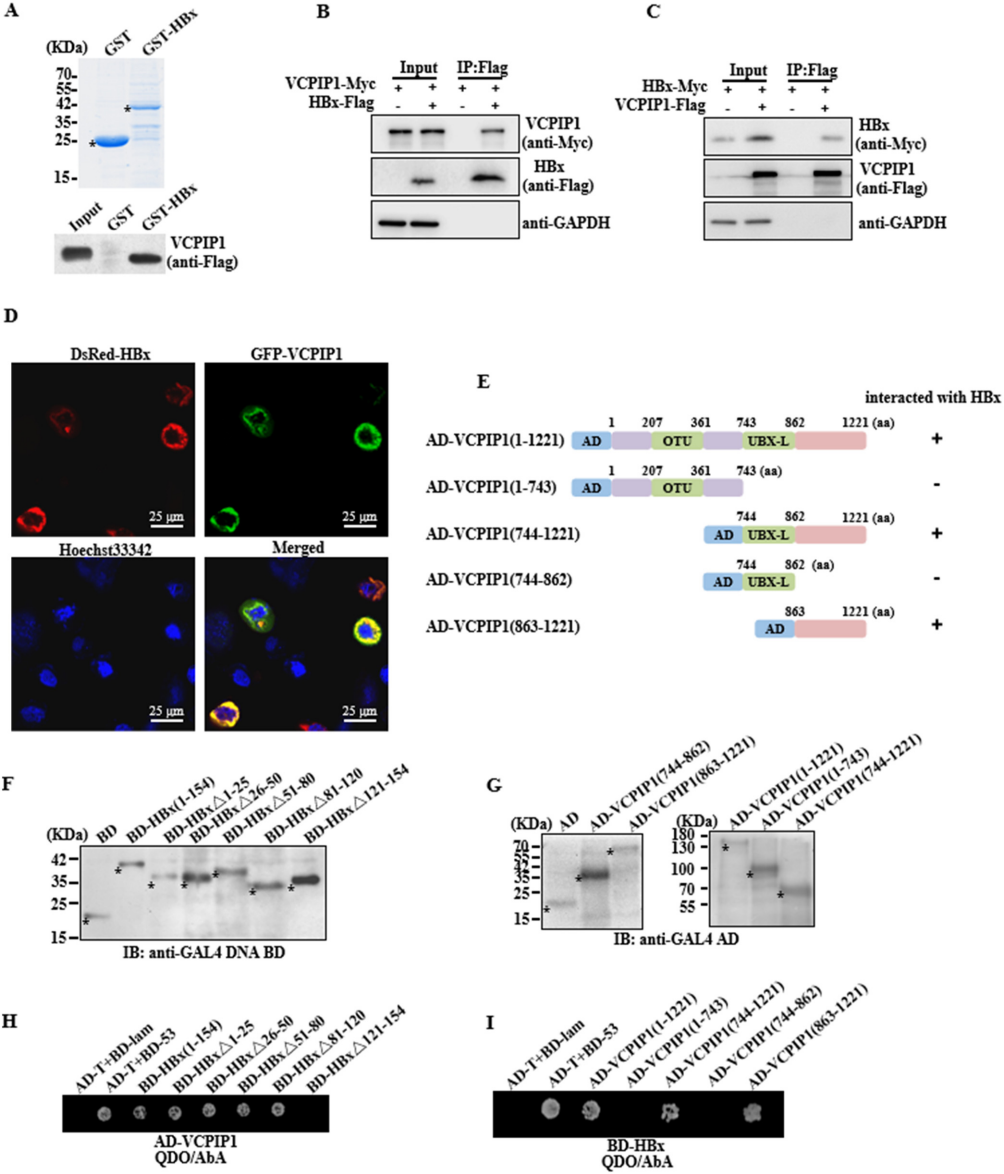
HBx interacted with VCPIP1. (A) GST pulldown assay. Coomassie blue-stained SDS-PAGE of purified GST and GST-HBx proteins in E. coli. The purified GST and GST-HBx proteins were incubated respectively with reticulocyte lysate-translated VCPIP1-Flag protein for *in vitro* GST pull-down assay. (B and C) Co-IP. Western blot analysis of immunoprecipitated lysates from Huh7 cells coexpressed with Myc-tagged VCPIP1 and Flag-tagged HBx and from Huh7 cells coexpressed with Myc-tagged HBx and Flag-tagged VCPIP1 or empty constructs. GAPDH, glyceraldehyde-3-phosphate dehydrogenase. (D) Confocal microscopy assay. Huh7 cells were coexpressed with DsRed-tagged HBx and GFP-tagged VCPIP1, followed by incubation with Hoechst 33342 to stain the nucleus. (E) Schematic representation of wild-type VCPIP1 and its truncated variants. Plus (+) and minus (−) signs indicate the presence or the absence of protein interactions. OTU, ovarian tumor domain; UBX-L, ubiquitin regulatory X (UBX)-like domain. (F and G) Western blotting. Yeast strain Y2HGold was transformed with pBD-HBx and its deletion mutant plasmids, and Y187 was transformed with pAD-VCPIP1 and its truncated mutant plasmids. The expression of HBx mutants was analyzed with GAL4 DNA-BD antibody (F), and the expression of VCPIP1 mutants was analyzed using GAL4 AD antibody (G) by Western blotting. Asterisks indicate the fusion proteins. (H and I) Yeast two-hybrid assay. QDO/AbA plate selection of diploid transformants was used to analyze the interaction between HBx and VCPIP1.

Attempting to identify the novel DUBs that may be involved in regulating HBx stability, we subjected a yeast two-hybrid assay comprised of a 74-DUB library to the bait of the HBx as we have previously described (18). In the DUB library (Table 1), MPN domain-containing protein (MPND), ubiquitin carboxyl-terminal hydrolase 22 (USP22), COP9 signalosome complex subunit 6 (COPS6), and VCPIP1 were four DUBs that may have the capability of interacting with HBx. VCPIP1 is required for Golgi and endoplasmic reticulum (ER) membrane fusion (19), and intracellular HBx is mainly distributed to the cytosol as a critical modulator of HBV-related HCC (20, 21). We then validated their interaction using *in vitro* and *in vivo* assays. The GST pulldown assay demonstrated that VCPIP1 bound to HBx directly (Fig. 1A) *in vitro*. The physical interaction was confirmed *in vivo* by Co-IP and confocal microscopy assays. Huh7 cells were transiently cotransfected with pVCPIP1-Myc and pHBx-Flag, or with empty vectors, and the Flag antibody-conjugated agarose beads were used for coimmunoprecipitation. VCPIP1 overexpression was significantly precipitated with HBx compared to vector transfection (Fig. 1B), and a reverse Co-IP experiment also validated the binding (Fig. 1C). A confocal microscopy assay showed that the DsRed-tagged HBx was colocalized with the green fluorescent protein (GFP)-tagged VCPIP1 within the cytosol of transfected Huh7 cells (Fig. 1D).

**TABLE 1:** Seventy-four DUBs used in the yeast-two hybrid assay^*a*^

Gene	GenBank no.	Length (bp)	MW (kDa)	CDS
USP2	BC002854.1	1,818	66.66	177…1994
USP3	NM_006537.3	1,563	57.31	230…1792
USP4	NM_003363.3	2,892	106.04	80…2971
USP5	NM_001098536	2,577	94.49	31…2607
USP6	NM_001304284.1	4,221	154.77	2231…6451
USP7	NM_003470.2	3,309	121.33	200…3508
USP8	NM_005154.4	3,357	123.09	204…3560
USP10	BC000263.1	2,397	87.89	114…2510
USP11	NM_001371072.1	2,763	101.2	36…2798
USP12	NM_182488	1,113	40.81	258…1370
USP13	NM_003940.2	2,592	95.04	82…2673
USP14	NM_005151.3	1,485	54.78	217…1701
USP15	NM_006313.3	2,859	104.83	12…2870
USP16	BC030777.2	2,469	90.53	159…2627
USP18	NM_017414	1,119	41.03	339…1457
USP19	BC146752.1	3,957	145.09	162. .4118
USP20	BC039593.1	2,742	100.54	159…2900
USP21	BC090946.1	1,698	62.26	326…2023
USP22	BC126898.1	1,542	56.54	1…1542
USP25	NM_001283041.2	3,378	123.86	461…3838
USP26	BC101189.2	2,742	100.54	51…2792
USP28	NM_001346258.1	3,138	115.06	70…3207
USP29	HQ258661.1	2,736	100.32	20…2755
USP30	AJ586136	1,554	56.98	1…1554
USP32	NM_032582.3	4,815	176.55	287…5101
USP33	NM_201626.2	2,487	91.19	375…2861
USP35	NM_020798.3	3,056	112.09	357…3413
USP36	BC071582.1	3,372	123.64	302…3673
USP37	BC112901.1	2,658	97.46	430…3087
USP38	BC068975.1	3,129	114.73	482…3610
USP39	NM_001256725	1,698	62.26	11…1708
USP41	AJ586979.1	921	33.77	1…921
USP43	NM_153210.4	3,372	123.64	97…3468
USP44	BC030704.1	2,139	78.43	126…2264
USP46	NM_022832.3	1,101	40.37	186…1286
USP48	NM_032236	3,108	113.96	193…3300
USP50	BC146493.1	1,020	37.4	35…1054
USP51	NM_201286.3	2,136	78.32	80…2215
USP54	NM_152586.3	5,055	185.35	18…5072
USPL1	BC038103.2	3,279	120.23	343…3621
USP17L2	NM_201402.2	1,593	58.41	1…1593
CYLD	NM_001042412.1	2,862	104.94	303…3164
PAN2	BC024043.1	3,597	131.89	220…3816
UCHL1	NM_004181.4	672	24.64	97…768
UCHL3	BC018125.2	693	25.41	31…723
UCHL5	NM_001199261.2	987	36.19	134…1120
BAP1	NM_004656.3	2,190	80.3	228…2417
OTUB1	NM_017670.2	816	29.92	605…1420
OTUB2	AY177201.1	705	25.85	1…705
OTUD1	NM_001145373.2	1,446	53.02	190…1635
OTUD4	NM_001102653.1	3,150	115.5	139…3288
OTUD6A	NM_207320.2	867	31.79	35…901
OTUD6B	AK291646.1	882	32.34	22…903
OTUD7B	NM_020205.3	2,532	92.84	357…2888
A20	NM_001166402.1	2,439	89.43	78…2516
FAM105B	NM_138348.5	897	32.89	182…1240
ZRANB1	NM_017580.2	2,127	77.99	372…2498
YOD1	NM_018566.3	1,047	38.39	48…1094
VCPIP1	NM_025054.4	3,669	134.53	260…3928
ATXN3	BC033711.1	1,113	40.81	13…1125
ATXN3L	BC137186.1	1,068	39.16	200…1267
JOSD1	NM_001360235.2	609	22.33	779…1387
JOSD2	NM_001270639.1	567	20.79	257…823
STAMBP	NM_213622.2	1,275	46.75	526…1800
STAMBPL1	NM_020799.3	1,311	48.07	506…1816
BRCC3	NM_024332.3	951	34.87	109…1059
COPS5	NM_006837.2	1,005	36.85	332…1336
COPS6	NM_006833.4	984	36.08	32…1015
EIF3H	BC090880.1	1,050	38.5	7…1056
EIF3S5	NM_003754.2	1,074	39.38	34…1107
PSMD7	NM_002811.4	975	35.75	141…1115
PSMD14	NM_005805.5	933	34.21	468…1400
MPND	BC032652.1	1,416	51.92	25…1440
MYSM1	NM_001085487.2	2,487	91.19	24…2510

a MW, molecular weight; CDS, coding sequence.

To determine the essential domains of HBx and VCPIP1 responsible for the interaction, a series of deletion mutants were constructed continuously at ~30 aa of HBx as previously described (16), or according to the basic structure of VCPIP1 (19), a series of truncated VCPIP1s were obtained as schematically illustrated in Fig. 1E. The expression of the HBx deletion mutants fused with the GAL4 DNA-binding (BD) domain, and the VCPIP1 truncation mutants fused with the GAL4 AD domain were detected (Fig. 1F and G). The HBx C-terminal sequence, spanning aa 121 to 154, predominated in the HBx-VCPIP1 binding, as seen in the colony abolishment in the yeast two-hybrid assay with pGBKT7-HBxΔ121–154, which lacked the sequences (Fig. 1H). When determining the interacting domain of VCPIP1 for HBx binding, it was found that the aa 863 to 1221 sequence at the C-terminal end was the essential domain (Fig. 1I).

### VCPIP1 is required for the increased HBx expression level with or without HBV.

**FIG 2:**
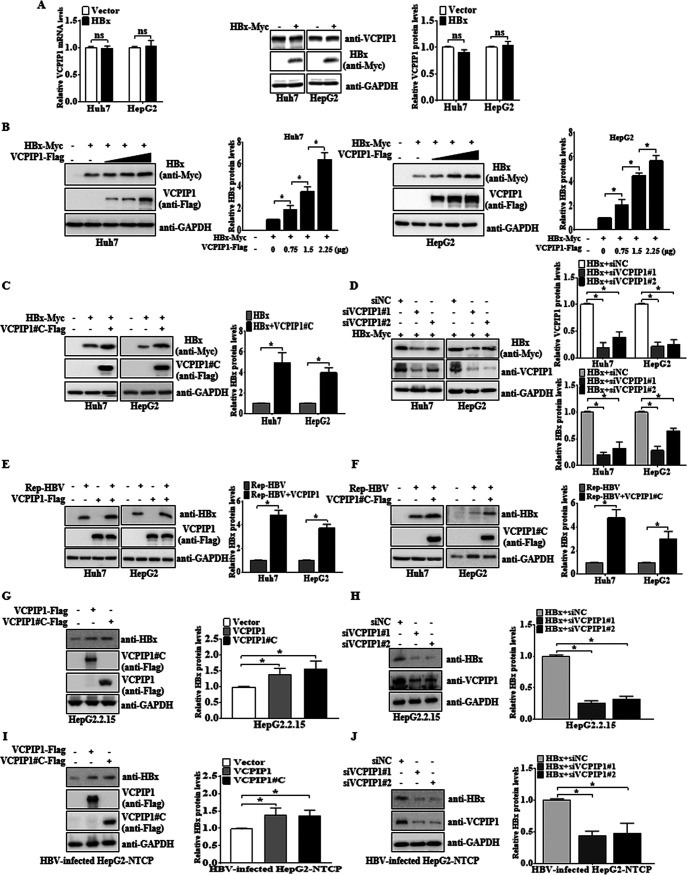
VCPIP1 expression increased HBx protein levels. (A) HBx expression had no effects on VCPIP1 mRNA and protein levels. VCPIP1 mRNA and protein levels from Huh7 and HepG2 cells transfected with pHBx-Myc or empty vector were analyzed by qRT-PCR and Western blotting, respectively. The relative mRNA and protein quantitative values of VCPIP1 were normalized to the corresponding GAPDH. *n* = 3; ns, not significant. (B) Western blot analysis of Huh7 and HepG2 cells cotransfected with pHBx-Myc and pVCPIP1-Flag. Values represent percentages of the HBx proteins normalized against GAPDH and compared to the signal of the HBx alone. (C) Western blot analysis of Huh7 and HepG2 cells cotransfected with Myc-tagged HBx plasmid and Flag-tagged VCPIP1 C-terminal (aa 863 to 1221) plasmid or empty vector. Values represent the percentages of the HBx proteins normalized against GAPDH and compared to the signal of HBx alone. (D) Western blot analysis of proteins isolated from cells cotransfected with pHBx-Myc and siVCPIP1#1 or siVCPIP1#2. Values represent the percentages of the HBx proteins normalized against GAPDH and compared to the siNC group. (E to J) Cell lysates were harvested with Western and IP lysis buffer and subjected to Western blotting to detect HBx in the context of HBV replication or infection using anti-HBx antibody (Abcam). (E and F) Western blot detection of HBx protein levels from cells transfected with 2 μg pRep-HBV harboring the 1.2-fold length of the HBV full genome alone or cotransfected with the wild-type VCPIP1 (aa 1 to 1221) (E) or VCPIP1 C-terminal end (aa 863–1221) (F). Bar graphs show the results of densitometric analysis of HBx proteins normalized against GAPDH and standardized to the pRep-HBV-only group. (G and H) Western blot analysis of HBV-encoded HBx protein from HepG2.2.15 cells transfected with the wild-type VCPIP1 (aa 1 to 1221) and VCPIP1 C-terminal end (aa 863 to 1221) (G) or siVCPIP1 against endogenous VCPIP1 (H). (I and J) HBx protein levels were detected from HepG2-NTCP cells infected with HBV particles expressing the wild-type VCPIP1 (aa 1 to 1221) or VCPIP1 C-terminal end (aa 863 to 1221) or siVCPIP1 by Western blotting. Values represent the percentages of the HBx proteins normalized against GAPDH and compared to the signal of the HBx. All of the values in the graph are means ± SD. *n* = 3; *, *P* < 0.05.

VCPIP1 expression in Huh7 and HepG2 cells was detected to demonstrate that both VCPIP1 mRNA and protein levels were not affected by HBx (Fig. 2A). Regarding the DUB nature of VCPIP1 in the OTU subfamily, we presumed that VCPIP1 would increase the HBx protein expression. As expected, ectopic VCPIP1 expression led to significantly increased HBx in a dose-dependent manner in both Huh7 and HepG2 cells (Fig. 2B). Notably, an identical effect was obtained by the VCPIP1#C plasmid transfection, which took the interacting domain of aa 863 to 1221 (Fig. 2C). VCPIP1-targeting small interfering RNA (siRNA) (siVCPIP1#1 and #2) treatment clearly demonstrated the decrease of HBx expression in comparison to negative-control siRNA (siNC) (Fig. 2D). To determine the effects of VCPIP1 on the HBx protein synthesis in the HBV replication-competent context, first, Huh7 and HepG2 cells were cotransfected along with pRep-HBV plasmid harboring a 1.2-fold length of wild-type HBV full genome. Both the full-length VCPIP1 and its C-terminal aa 863 to 1221 upregulated the HBx expression induced by plasmid containing replication-competent full-length HBV (Fig. 2E and F). Then, the similar effects of VCPIP1 and its C-terminal truncation increasing the HBx expression were validated in HepG2.2.15 cells (Fig. 2G), but HBx was reduced by silencing endogenous VCPIP1 (Fig. 2H). Whether VCPIP1 affected the HBx protein stability in the context of HBV infection was tested ultimately in the HepG2-NTCP-reconstituted hepatoma cells infected with HBV particles harvested from HepAD38 cells (Fig. 2I and J). Taken together, these results suggest that VCPIP1 is indispensable for HBx expression with or without the presence of infectious HBV particles.

### VCPIP1 increases HBx stability.

**FIG 3:**
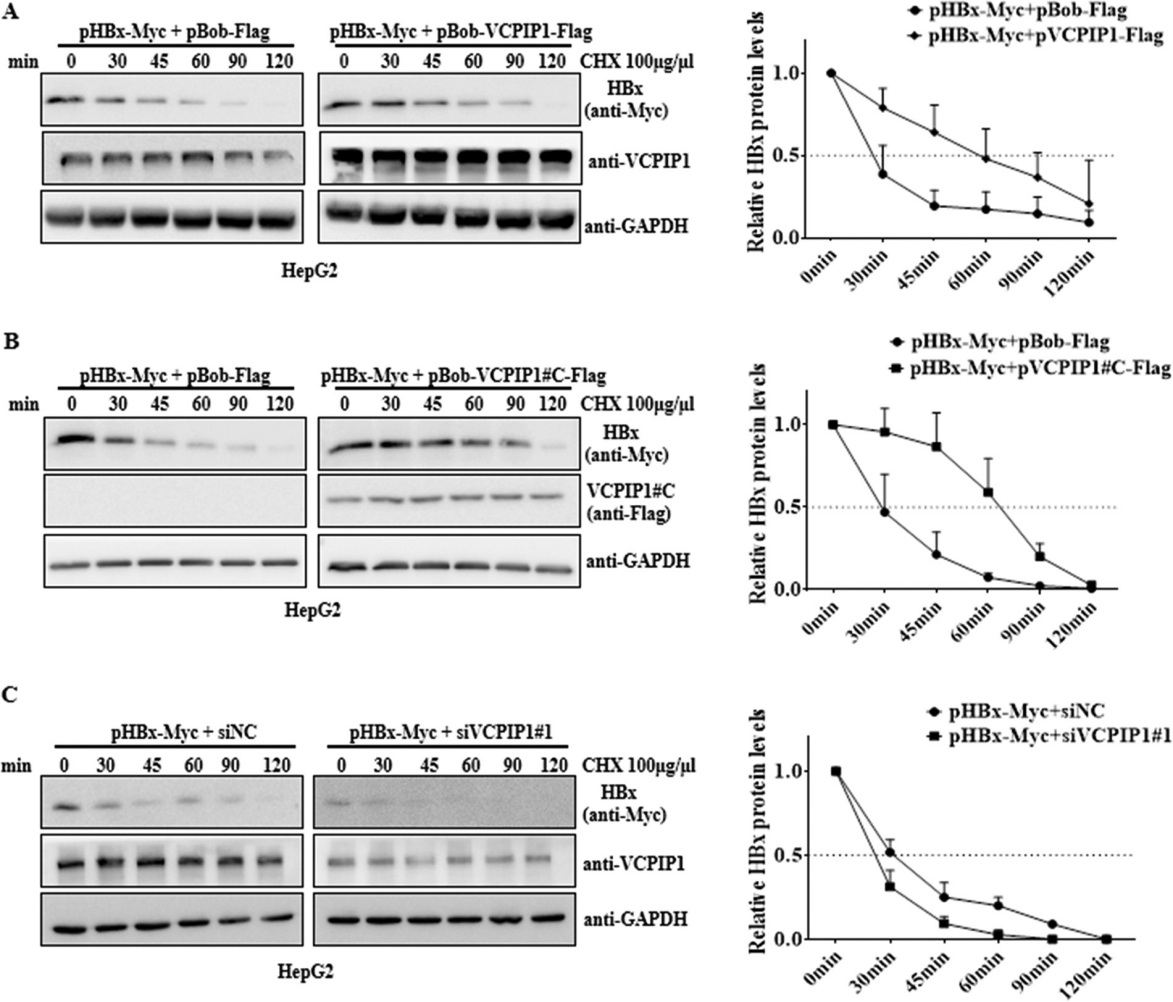
VCPIP1 increased HBx stability. (A and B) HepG2 cells expressed Myc-tagged HBx alone or coexpressed full-length VCPIP1-Flag (A) or C-terminal VCPIP1-Flag (aa 863 to 1221) (B). After 48 h, the cells were incubated with CHX for different times. Extracts were examined with anti-Myc antibody. (C) HepG2 cells were transfected with pHBx-Myc alone or cotransfected with siVCPIP1#1 or siNC and then treated with CHX. The HBx protein levels were analyzed with anti-Myc antibody. The HBx expression intensity for each time point was quantified by densitometry and plotted. Quantification of the remaining HBx level of each group shown in the right graph was normalized to the corresponding GAPDH and then to the signal at 0 min. Values in the graph are means ± SD. *n* = 3.

To verify the VCPIP1-induced increase in the HBx protein levels, we used cycloheximide (CHX) to block protein synthesis to determine whether VCPIP1 altered HBx stability. VCPIP1 overexpression significantly extended the half-life of HBx protein from 30 min to 60 min, either upon the full-length VCPIP1 protein or the aa 863 to 1221 interacting domain (Fig. 3A and B). In contrast, inhibiting VCPIP1 resulted in the half-life of HBx being reduced from 30 min to 20 min (Fig. 3C). These results demonstrate that the interaction between HBx and VCPIP1 may increase HBx stability.

### VCPIP1 inhibits HBx degradation through the proteasome pathway in a ubiquitin-independent manner.

**FIG 4:**
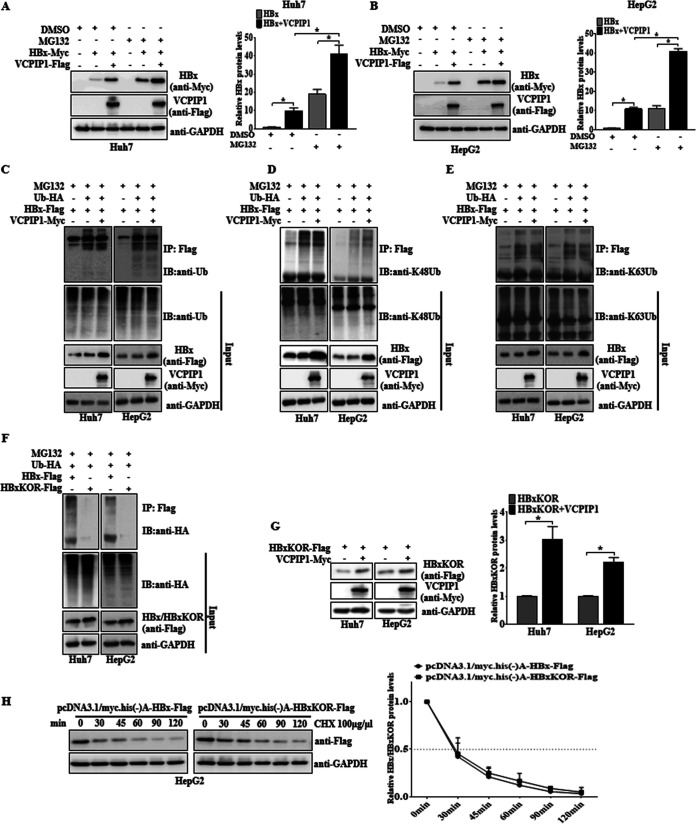
VCPIP1 inhibited the HBx degradation through the proteasome pathway in a ubiquitin-independent manner. (A and B) VCPIP1 mediated HBx stability through the proteasomal pathway. Huh7 (A) and HepG2 (B) cells were transfected with Myc-tagged HBx with or without Flag-tagged VCPIP1. After transfection, the cells were treated with MG132 or an equal volume of dimethyl sulfoxide (DMSO). The signal intensities of HBx were normalized to GAPDH and then compared to that of the DMSO-treated HBx expression-only group. (C to E) Ubiquitination assay for detecting ubiquitinated HBx. Huh7 and HepG2 cells were cotransfected with Flag-tagged HBx and HA-tagged ubiquitin (Ub), with or without Myc-tagged VCPIP1, and then treated with MG132. The HBx proteins were precipitated by anti-Flag-conjugated agarose beads, and the ubiquitinated HBx was detected by anti-ubiquitin antibody (C), anti-K48 ubiquitin antibody (D), and anti-K63 ubiquitin antibody (E). IB, immunoblot. (F) Ubiquitination assay for mutated HBx. Huh7 and HepG2 cells were cotransfected with HA-tagged ubiquitin and Flag-tagged HBx or Flag-tagged HBxKOR (all ubiquitin recognition sites of lysines in the HBx protein were mutated to arginine) and then treated with MG132. The cell lysates were harvested and subjected to the ubiquitination assay followed by immunoblotting with anti-HA antibody. (G) Huh7 and HepG2 cells were cotransfected with Flag-tagged HBxKOR with or without Myc-tagged VCPIP1. The protein levels of HBxKOR were analyzed with anti-Flag antibody. The graph shows the signal intensities of HBxKOR cotransfected with VCPIP1, which were normalized by GAPDH and compared with the HBxKOR-only group. *n* = 3; *, *P* < 0.05. (H) HepG2 cells were transfected with pHBx-Flag or pHBxKOR-Flag and then treated with CHX for different times. Anti-Flag antibody was used to analyze the wild-type or mutant HBx proteins. The wild-type or mutant HBx expression intensities for each time point were quantified by densitometry and plotted. Values in the graph are the means ± SD. *n* = 3.

DUBs usually prevent substrate degradation by deubiquitination through the ubiquitin-dependent proteasome pathway (15). pHBx-Myc was cotransfected with pVCPIP1-Flag or the empty vectors into Huh7 cells, which were subsequently treated with the proteasome inhibitor MG132. Treatment with MG132 greatly enhanced the HBx protein expression especially in combination with the ectopic VCPIP1 expression (Fig. 4A). A similar effect was observed in HepG2 cells (Fig. 4B). To determine whether the increased HBx expression by VCPIP1 was due to the decreased ubiquitination, we performed an *in vivo* ubiquitination assay by detecting the ubiquitination of HBx proteins using the Flag antibody-conjugated agarose beads to precipitate the HBx. Although VCPIP1 overexpression led to significantly increased HBx in the Huh7 and HepG2 cells, it did not redice the amounts of HBx ubiquitination (Fig. 4C). It is well known that K48- and K63-linked ubiquitination chains are the most abundant chain types, and K48-linked ubiquitination often leads to the degradation of target proteins by the 26S proteasome (22). Next, we determined whether VCPIP1 influenced the K48-Ub (ubiquitin) and K63-Ub (ubiquitin)-induced ubiquitination of HBx. The results revealed that HBx expression alone could induce K48- and K63-linked polyubiquitination, whereas coexpression of VCPIP1 with HBx had no effects on the K48- (Fig. 4D) and K63-linked (Fig. 4E) polyubiquitination of HBx. To further validate VCPIP1 regulation of HBx in a ubiquitin-independent manner, we constructed a pHBxKOR-Flag plasmid, in which all lysine residues for HBx ubiquitination were replaced with arginine. We verified the compromised capability of the mutant pHBxKOR-Flag to be ubiquitinated compared to that of the wild-type HBx (Fig. 4F). However, we found that ectopic VCPIP1 expression nevertheless promoted the expression of the ubiquitination-lacking HBx mutant (Fig. 4G). Cycloheximide experiments were performed and indicated that the HBxKOR mutant proteins appeared to be as stable as the wild-type HBx with an estimated half-life of 30 min in HepG2 cells (Fig. 4H). Taken together, these results indicate a novel mechanism by which VCPIP1 stabilizes the HBx protein via the ubiquitin-independent proteasome pathway.

### VCPIP1 recruits PSMC3 to form a larger ternary complex with HBx.

**FIG 5:**
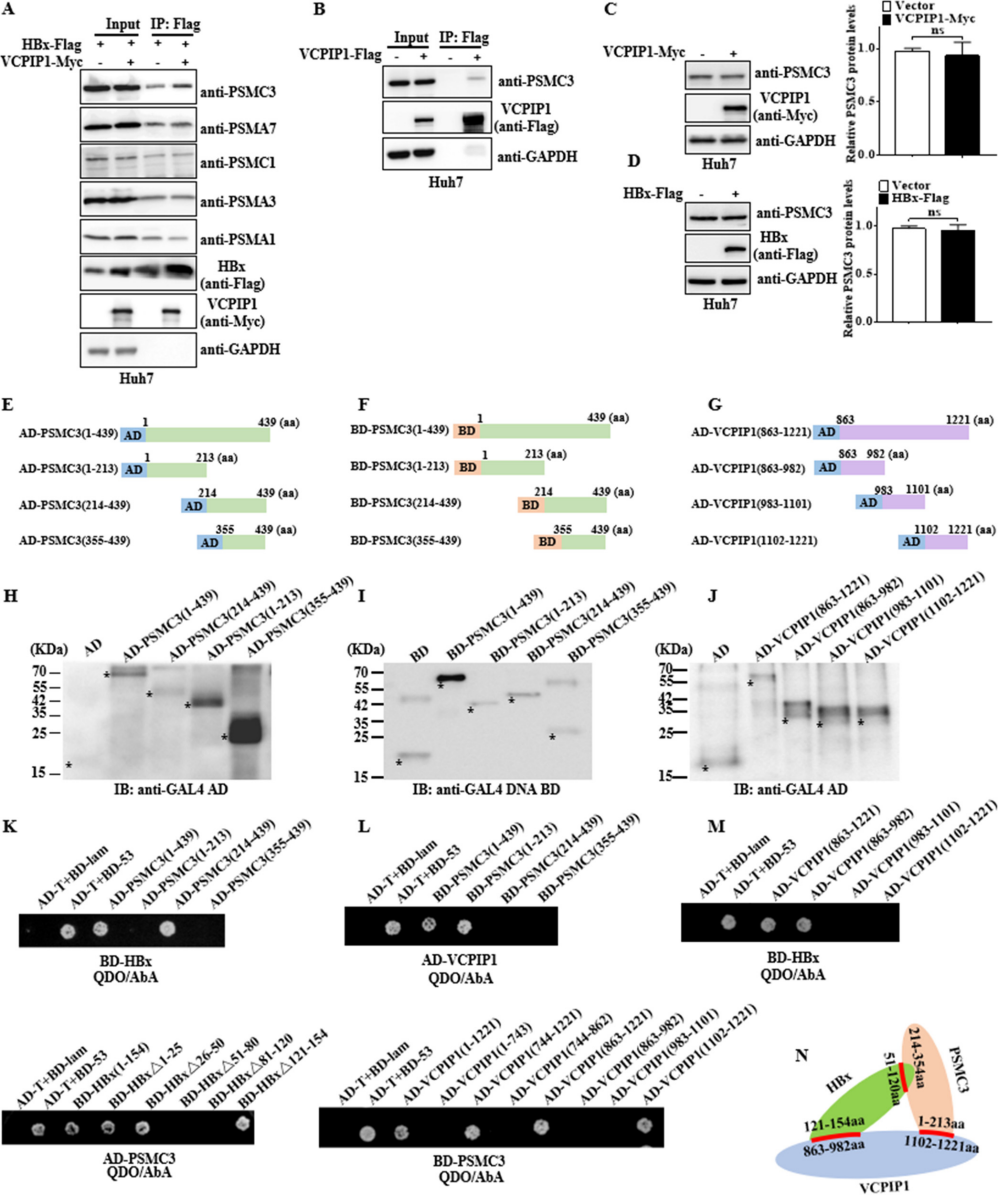
VCPIP1 recruited PSMC3 to form a larger ternary complex with HBx. (A) Huh7 cells were transfected with pHBx-Flag alone or cotransfected with pVCPIP1-Myc and then treated with MG132. The extracts were immunoprecipitated by the Flag antibody-conjugated agarose beads, and the immunocomplex was analyzed by Western blotting. (B) Huh7 cells were transfected with pVCPIP1-Flag, and the corresponding empty vectors were used as the control. Intracellular PSMC3 was detected in the immunoprecipitated complex of Flag-tagged VCPIP1 by Western blotting with anti-PSMC3 antibody. (C and D) Western blot analysis showed that endogenous PSMC3 levels in Huh7 cells were unchanged with transfection of pVCPIP1-Myc or pHBx-Flag compared with that of empty vector. *n* = 3; ns, not significant. (E to G) Schematic diagrams of the full-length and truncated PSMC3 mutants fused to AD epitope (E) or DNA-BD epitope (F) at the N-terminal end and VCPIP1#C truncated mutants fused to AD epitope (G). (H and I) Western blot analysis using GAL4 AD antibody (H) or GAL4 DNA-BD antibody (I) on fusion proteins from AD-PSMC3 and DNA BD-PSMC3 recombinant plasmids which were transformed into Y187 or Y2HGold yeast. Asterisks indicate each fusion protein. (J) Western blotting was used to analyze the VCPIP1 fusion proteins using GAL4 AD antibody. (K to M) Yeast two-hybrid analysis of the interactions between HBx-PSMC3 proteins (K), between VCPIP1-PSMC3 proteins (L), and between HBx and the truncated VCPIP1#C (M). (N) Schematic illustration of HBx, VCPIP1, and PSMC3 proteins binding to form a ternary complex.

Degradation of intracellular proteins is principally mediated by the 26S proteasome, which consists of two 19S regulatory complexes and a 20S proteolytic core proteasome (23). Considering that VCPIP1 stabilized HBx protein through the proteasome pathway and that we previously identified 20S proteasome subunit PSMA7-mediated degradation of HBx (24), we investigated if VCPIP1 could hijack the same mechanism. As previous reports have indicated the association of HBx with multiple subunits of the 26S proteasome, including PSMA1, PSMA3, PSMA7, PSMC1, and PSMC3 (9, 13), we performed a Co-IP-based screening to identify the key component from them that may specifically respond to VCPIP1. Notably, only the enrichment of PSMC3 was identified in the binding of HBx under the context of VCPIP1 overexpression, while PSMC1, PSMA1, PSMA3, and PSMA7 were not responsive (Fig. 5A). Furthermore, the Co-IP assay detected endogenous PSMC3 expression with the VCPIP1 overexpression but not with the empty vector control (Fig. 5B), and the interaction did not result from the possible increase of PSMC3 by VCPIP1 (Fig. 5C) and HBx (Fig. 5D). Here, we demonstrate PSMC3, which belonged to the 19S regulator of the 26S proteasome (23), as the interacting partner of VCPIP1. The mutual interactions between VCPIP1 and HBx (Fig. 1), HBx and PSMC3 (Fig. 5A), and PSMC3 and VCPIP1 (Fig. 5B) were identified. Next, we attempted to determine the binding pattern between them. In the schematic illustrations in Fig. 5E and F, we prepared a series of truncation plasmids of the PSMC3 (25) by fusing the individual fragments to the pGADT7 or pGBKT7 vector. All of the truncated PSMC3 proteins were expressed well in yeast (Fig. 5H and I). In addition, to further map the exact domain in the VCPIP1 C-terminal aa 863 to 1221 to account for the mutual interaction, three truncations at approximately 120 aa were constructed for pGADT7 (Fig. 5G) and the fusion protein expressions were detected by Western blotting (Fig. 5J). Then, yeast two-hybrid assays were performed to explore the interaction pattern. The results demonstrated that PSMC3 aa 214 to 354 and HBx aa 51 to 120 were greatly important domains for HBx-PSMC3 interaction (Fig. 5K). Similarly, PSMC3 aa 1 to 213 and VCPIP1 aa 1102 to 1221 were essential for their interaction (Fig. 5L). Combined with the earlier results demonstrating that HBx aa 121 to 154 and VCPIP1 aa 863 to 1221 were required for VCPIP1-HBx interaction (Fig. 1H and I), we narrowed down the sequence for interacting with HBx to VCPIP1 C-terminal aa 863 to 982 (Fig. 5M). Collectively, we propose a mutual interaction working model between VCPIP1, PSMC3, and HBx, in which they form a large ternary complex through VCPIP1 C-terminal-recruited binding of PSMC3 to HBx (Fig. 5N).

### VCPIP1-recruited PSMC3 stabilizes the HBx protein efficiently.

**FIG 6:**
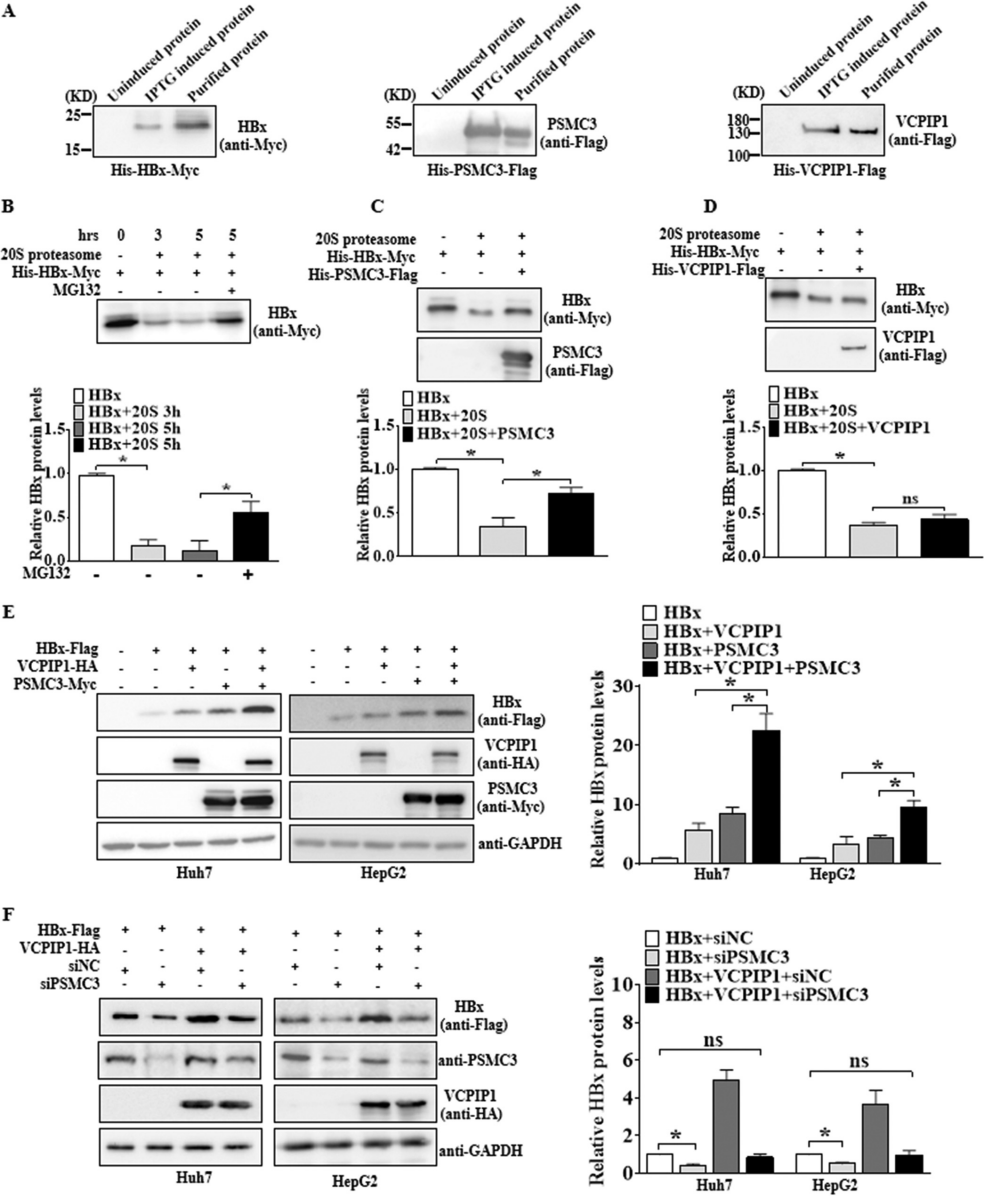
VCPIP1-recruited PSMC3 stabilized the HBx protein efficiently. (A) Western blot detection of nickel affinity-purified recombinant proteins. Recombinant plasmids of His-tagged HBx with Myc epitope, His-tagged PSMC3 with Flag epitope, or His-tagged VCPIP1 with Flag epitope were transformed into competent E. coli DE3 cells and then induced with or without isopropyl-β-d-thiogalactopyranoside (IPTG). The cells were harvested and ultrasound lysed to obtain the supernatant. The lysates were purified with nickel-chelating beads at 4°C overnight and then examined by Western blotting with anti-Myc or anti-Flag antibodies. (B) Western blot analysis of *in vitro*-purified His-tagged HBx with Myc epitope incubated with or without 20S proteasome; 20S proteasome inhibition by MG132 was used as a control. (C and D) Western blot analysis of HBx stability of *in vitro*-purified His-tagged HBx with Myc epitope and purified His-tagged PSMC3 with Flag epitope (C) or purified His-tagged VCPIP1 with Flag epitope (D) incubated with or without 20S proteasome. The relative HBx protein levels were obtained by comparison with the groups of those treated without PSMC3 or VCPIP1. *n* = 3; *, *P* < 0.05; ns, not significant. (E) PSMC3 synergized the effects of VCPIP1 overexpression on HBx protein level *in vivo*. Huh7 and HepG2 cells were transfected with pHBx-Flag alone or cotransfected with pVCPIP1-HA, pPSMC3-Myc, or all three. Then, the cells were lysed and harvested for Western blotting with anti-Flag antibody to analyze the presence of HBx. (F) Silencing PSMC3 downregulated HBx protein levels via VCPIP1. Huh7 and HepG2 cells were cotransfected with Flag-tagged HBx, HA-tagged VCPIP1, and siPSMC3 targeting endogenous PSMC3, or siNC as the control. Then, the cells were harvested for Western blotting with anti-Flag antibody to assess HBx expression. The relative HBx protein levels of those affected by PSMC3 or VCPIP1 were obtained by normalization to GAPDH and comparison with the HBx-expressed-only group. *n* = 3; *, *P* < 0.05; ns, not significant.

The proteasome plays a central role in degrading the majority of intracellular proteins in the eukaryotes, among which the 20S proteasome catalytic particle is specifically involved in degrading nonubiquitinated proteins that are naturally unfolded (26). Some studies have indirectly demonstrated that HBx is a loosely structured viral protein (27, 28). We tested whether HBx could undergo proteasome degradation in a ubiquitin-independent manner by using purified 20S proteasome in an *in vitro* assay. The His-tagged HBx-Myc recombinant protein was induced and purified from Escherichia coli and detected by Western blotting (Fig. 6A). HBx was decreased significantly following incubation with the 20S proteasome (Fig. 6B) but was rescued by MG132 treatment, which inhibited the direct degradation of HBx by the 20S proteasome. To determine if the 20S proteasome was the functional component for counteracting HBx stability via VCPIP1-mediated PSMC3, purified VCPIP1-Flag and PSMC3-Myc proteins were also prepared, and both were tagged with 6× His epitope (Fig. 6A). It was determined that the addition of PSMC3 *in vitro* inhibited the HBx degradation induced by the 20S proteasome (Fig. 6C), while the addition of VCPIP1 had no such effect (Fig. 6D). As the *in vitro* system lacked the integral proteasome, we detected HBx expression with VCPIP1 and PSMC3 cotransfection in cells and found a consistent increase in HBx induced by ectopic PSMC3 expression upon VCPIP1 overexpression (Fig. 6E). RNA interference (RNAi) targeting endogenous PSMC3 was used to validate that PSMC3 was required for VCPIP1-mediated HBx stability but was not a contributing factor. More importantly, it was clearly shown that siPSMC3 treatment greatly impaired the HBx expression increased by VCPIP1 overexpression compared to the siRNA control (Fig. 6F). Collectively, the results indicate that VCPIP1-recruited PSMC3 associating with HBx functionally prevents the HBx degradation by the 20S proteasome.

### VCPIP1-stabilized HBx functions as a canonical transactivator and inhibits cell proliferation.

**FIG 7:**
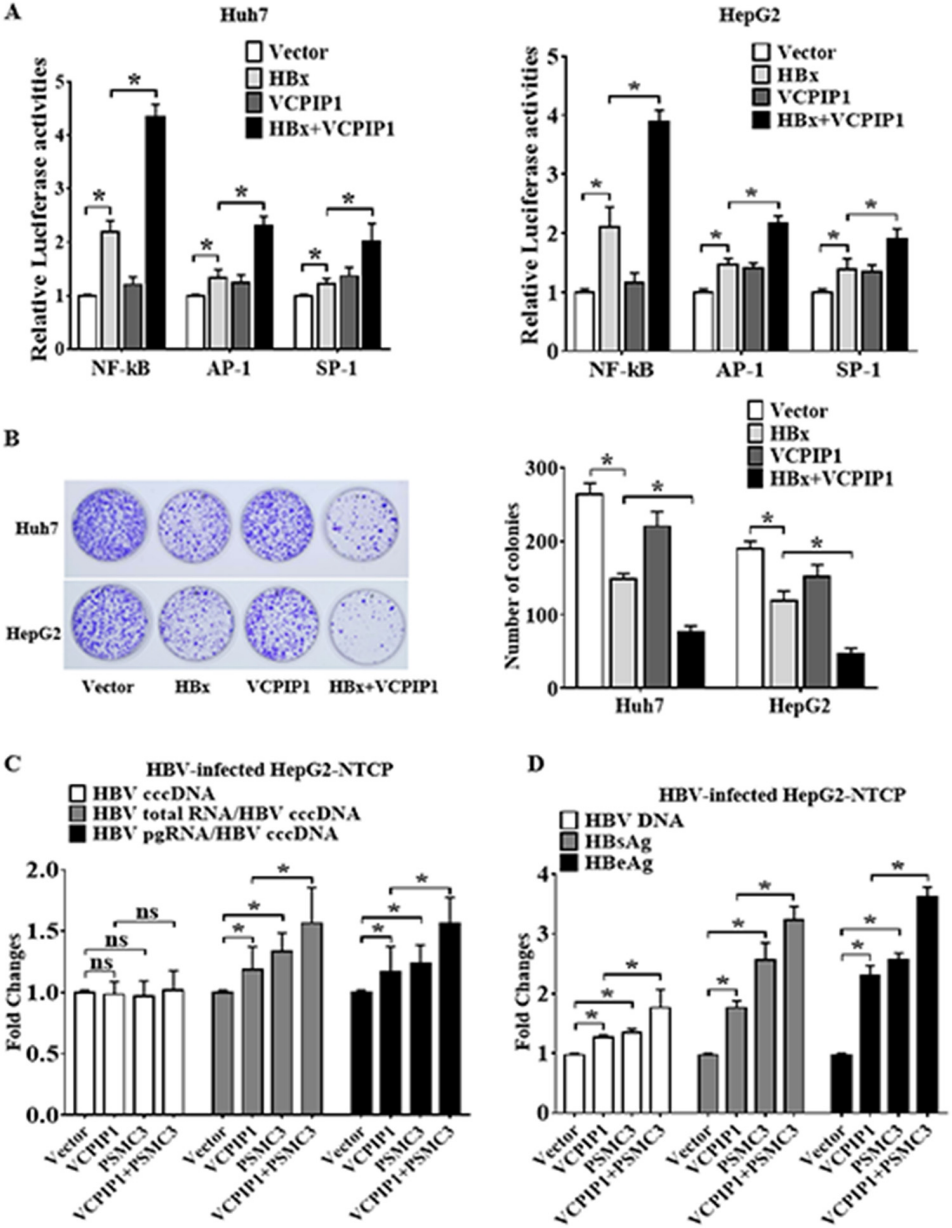
VCPIP1-HBx interaction facilitated the HBx protein to exert its pleiotropic effects. (A) VCPIP1-stabilized HBx functioned as a canonical transactivator and inhibition of cell proliferation. Dual-luciferase reporter assay of Huh7 and HepG2 cells cotransfected with NF-κB, AP-1, or SP-1 reporter plasmids and *Renilla* luciferase internal control plasmids. Luciferase activities were normalized with *Renilla* luciferase values and then standardized by the empty vector group. *n* = 3; *, *P* < 0.05. (B) Colony formation assay. Huh7 or HepG2 cells were cotransfected with pHBx-Flag, pVCPIP1-Myc, or both and then treated with G418. Twenty-one days later, the cells were stained by crystal violet. Bar graph shows the colony counts. Results are the means ± SD. *n* = 3; *, *P* < 0.05. (C) HepG2-NTCP cells were infected with HBV particles and then transfected with vector expressing VCPIP1-Flag, PSMC3-Myc, or both for 48 h. HBV cccDNA, total HBV RNA, and pgRNA were extracted and analyzed by real-time quantitative PCR (qPCR) with specific primers. The relative ratios were used to elevate HBV cccDNA transcription. (D) HBV DNA, HBsAg, and HBeAg in the HepG2-NTCP cell culture supernatant were quantified by qPCR and ELISA. *n* = 3; *, *P* < 0.05; ns, not significant.

One of the most striking functions of HBx is to stimulate the transcription of HBV and cellular transcriptional regulatory elements in the hepatocytes, thus promoting the progression of HCC (29). Here, we verified the transactivating effects of VCPIP1-stabilized HBx on the promoter activity of NF-κB, AP-1, and SP-1. The dual-luciferase reporter assay showed that further VCPIP1 expression significantly enhanced the HBx-induced transactivation activity of NF-κB, AP-1, and SP-1 in the Huh7 and HepG2 cells, whereas VCPIP1 alone had no such effects (Fig. 7A). Consistent with previous reports that HBx inhibits the clonal outgrowth of cells (30), we confirmed the inhibitory effect of HBx and stronger function of VCPIP1-stabilized HBx. HBx overexpression led to a dramatic decrease in the colony numbers of both Huh7 and HepG2 cells, and further involvement by VCPIP1 plasmid cotransfection resulted in an even larger inhibitory effect on cell proliferation by HBx (Fig. 7B).

### VCPIP1-recruited PSMC3 stabilizes HBx and promotes HBV cccDNA transcription in HBV-infected HepG2-NTCP cells.

Previous studies have indicated that the HBx proteins play a critical role in the HBV replication and cccDNA transcription (31). We next investigated the effects of VCPIP1-recruited PSMC3 on HBV cccDNA transcription in HBV-infected HepG2-NTCP cells. Real-time quantitative PCR was used to measure the level of HBV cccDNA and the total HBV RNA and pregenomic RNA (pgRNA). The ratio of total HBV RNA to cccDNA and that of pgRNA to cccDNA were calculated to evaluate cccDNA transcriptional activities. It was found that VCPIP1 or PSMC3 overexpression significantly increased the ratios of total HBV RNA to cccDNA and HBV pgRNA to cccDNA but did not alter HBV cccDNA levels, and the coexpression of VCPIP1 and PSMC3 had a more significant effect (Fig. 7C), suggesting HBV cccDNA transcription was increased. As has been reported previously, HBx could enhance HBV gene expression from episomal cccDNA (7). Consistently, overexpression of VCPIP1 or PSMC3 resulted in the HBV DNA level increase as well as HBsAg and HBeAg secretion (Fig. 7D). These results indicate the VCPIP1-recruited PSMC3 stabilized HBx and facilitated HBV cccDNA transcription.

**FIG 8:**
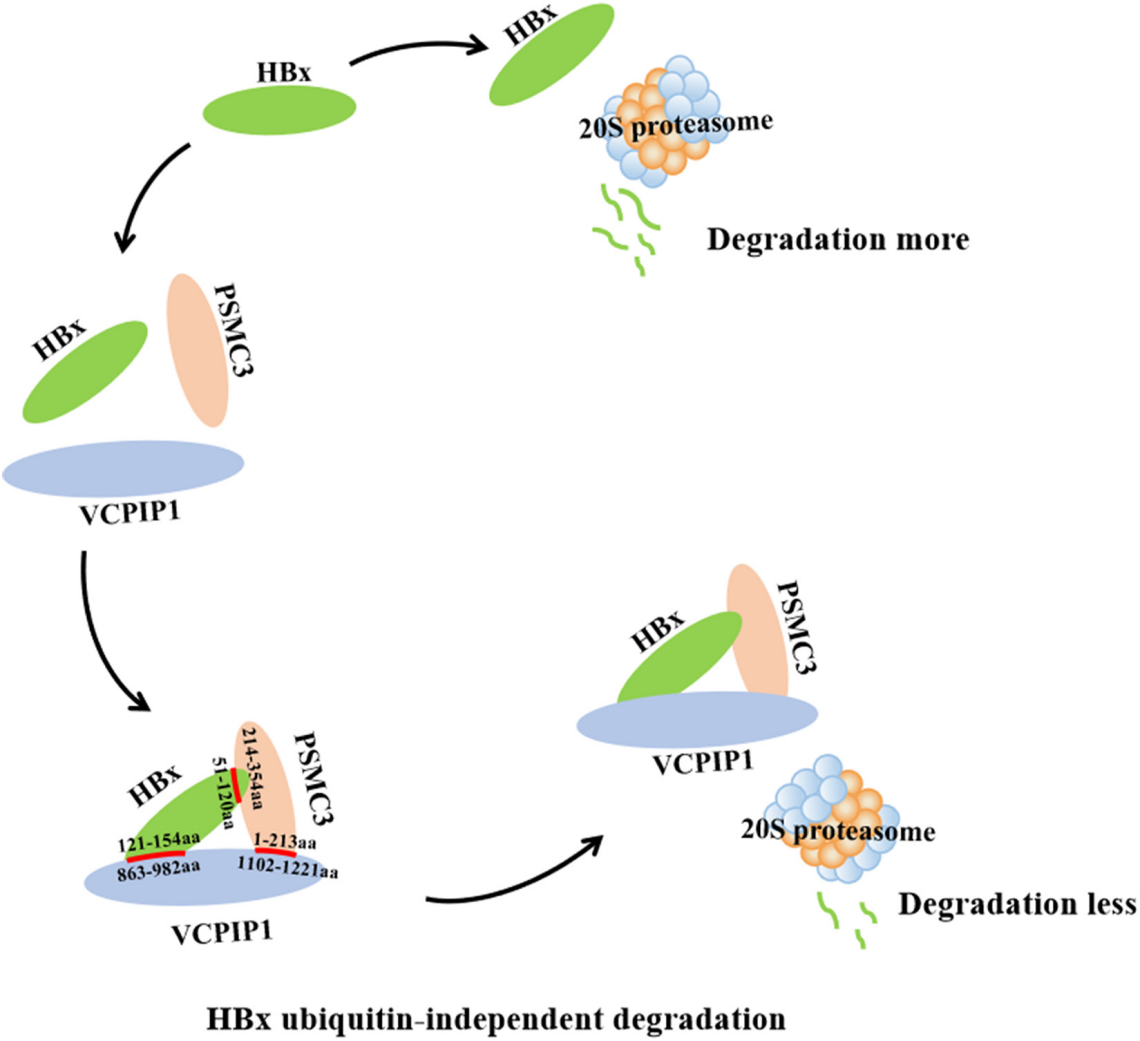
Proposed model for the role of VCPIP1 in regulating HBx protein levels. VCPIP1 interacts with HBx and PSMC3, leading to the inhibition of HBx degradation by the 20S proteasome.

Finally, Fig. 8 depicts a proposed working model indicating the interaction between VCPIP1, HBx, and PSMC3, in which VCPIP1 functions as a chaperone protein of HBx and can recruit PSMC3 also. Notably, PSMC3 exerts its effect independently from being a part of 19S complex to cooperate in blocking HBx proteasomal degradation.

## DISCUSSION

High HBx expression levels are a key factor in the pathogenesis of the HBV-related HCC (32, 33). The mechanism by which high HBx expression is sustained in HCC tissues remains largely unknown. However, HBx is usually recognized as a short-lived protein with a half-life of about 30 min, whose instability is attributable to proteasome pathway-mediated rapid degradation (34, 35). Posttranslational ubiquitin modification of the target protein leaves it vulnerable to 26S proteasome-mediated degradation, and the process is dynamic and reversible, which is orchestrated and precisely controlled via ubiquitinating enzymes and DUBs (36). The types of ubiquitinating enzymes and DUBs and their direct roles in the process require further investigation. To date, several E3 ubiquitin ligases, e.g., Siah-1 (12), murine double minute 2 (MDM2) (37), tripartite motif-containing 21 (TRIM21) (38), and mitochondrial E3 ubiquitin ligase (MARCH5) (39), degrade HBx, while USP15 has been identified as a DUB that stabilizes HBx (16). DUBs cleave ubiquitin molecules from either the E3 ligase or the target proteins, thereby preempting ubiquitin-mediated regulation (15). A plethora of DUBs are overexpressed in the HCC tissues and correlate significantly with poor clinical outcomes (40). Therefore, the host-virus cross talk between DUBs and HBx is fundamentally important and clinically relevant. In the present study, we identify a new DUB, VCPIP1, responsible for the HBx stability. First, we reported that VCPIP1 bound directly to HBx through its C-terminal aa 863 to 1221, and the interacting sequences were sufficient for HBx stability even in the absence of its N-terminal enzymatic activity. This ubiquitin-independent regulation by VCPIP1 prompted us to find other possible downstream effectors in the process. We determined that PSMC3, the subunit of the 19S regulatory complex of 26S proteasome in the UPS, was the candidate recruited by VCPIP1 to form a ternary complex with HBx through mutual interaction, which may structurally hinder HBx being transferred into the 26S proteasome.

VCPIP1 encodes a 1,221-aa DUB belonging to the OTU subfamily, and its N-terminal OTU enzyme activity is necessary for cleaving polyubiquitin chains from the target proteins (41), and it can also cleave monoubiquitin from DNA-dependent metalloprotease SPRTN (42). VCPIP1 was initially identified as a p47 interactor involved in dissociating the p97-p47-syntaxin 5 complex that critically affected membrane fusion (19). And the OTU enzyme activity of VCPIP1 is required for dissociating the p97-p47 complex binding to syntaxin 5 disassembly independent of proteasome-mediated proteolysis, resulting in the formation of a transient quaternary complex that can be dissociated during the cell cycle (43). A recent study found that phosphorylating VCPIP1 at S130 inhibited its deubiquitinase activity important for Golgi membrane fusion (44). Interestingly, Uchiyama et al. have reported a novel membrane fusion pathway in which VCPIP1 is also required for ER biogenesis through the dissociated p97-p37 complex; however, its deubiquitinating activity is unnecessary for p97-p37-mediated activity (45). These reports suggest that VCPIP1 has other functions apart from a deubiquitination role. Here, we demonstrate a novel mechanism by which VCPIP1 acts as a scaffold to the HBx protein, independent of its N-terminal enzymatic function domain. We sought to understand the stabilization effect exerted by VCPIP1 on HBx, especially in the absence of the DUB activity, and we discovered a novel role of the PSMC3 recruited by VCPIP1 to stabilize HBx instead of degrading it. Overall, VCPIP1 is a new DUB for HBx protein stability but not via deubiquitination, thereby broadening knowledge of the pathogenic involvement of the DUB family in viral infectious diseases.

PSMC3 is an ATPase subunit of the 19S regulatory complex of the 26S proteasome apparently involved in cellular events that do not require only protein hydrolysis; that is, PSMC3 overexpression appears not to cause general effects on proteasome function; rather, it appears to affect only specific targets, such as inhibiting the degradation of the tumor suppressor ARF (46), and with opposite effects, i.e., promoting the degradation of hypoxia-inducible factor 1 alpha (HIF1α) (47). Although many 26S proteasome subunits could bind with HBx, the underlying mechanisms and the *in vivo* consequences remain controversial (9). Here, we demonstrate that HBx has been identified as the substrate of PSMC3, whose binding is recruited by VCPIP1 in the cytoplasm to form ternary complexes. Barak et al. indicated a functional association of PSMC3 with HBx promoting HBV transcription in the nucleus, but the underlying mechanisms remained controversial (48). The present study suggested a different mechanism of PSMC3 and VCPIP1 cooperating directly to regulate HBx proteasomal degradation and then regulate HBV cccDNA transcription. Additionally, our study also shows a different mechanism regarding the other components of the 26S proteasome related to HBx stability. PSMA3 is recruited by Id-1 to interact with HBx and lead to its proteasomal degradation in a ubiquitin-dependent manner (13). PSMA7 interacts with the chromatin remodeling factor BAF155 to stabilize HBx in a ubiquitin-independent manner (24).

The lid of 19S consists of DUBs and ubiquitin receptors (49). However, whether DUBs are directly involved in 19S function in HBV pathogenesis is unknown. Here, we found that VCPIP1 associated with proteasomal subunits and with HBx and prevented HBx degradation. Another interesting result was that PSMC3 protein alone counteracted the 20S proteasome-induced HBx degradation *in vitro* system independent of the integral 19S complex, highlighting the effect of intracellular free PSMC3. Regarding PSMC3 stabilization of the cellular protein ARF, the intrinsically unstructured form of ARF *in vivo* renders it susceptible to degradation. However, binding with free PSMC3 causes the folding of ARF and renders it a poor substrate for 20S proteasome degradation (46). HBx is also a natively unstructured protein and may fold under specific conditions (27, 28). We propose that the property is stabilized by VCPIP1 recruiting PSMC3 to compress the HBx structure through the ternary binding pattern.

Furthermore, VCPIP1-HBx interaction appears to facilitate HBx protein to exert its pleiotropic effects including canonical transactivational activity and cell proliferation inhibition, suggesting its potential role in the pathogenesis of HBV-related hepatocellular carcinoma. Moreover, VCPIP1 recruited PSMC3, promoting HBx protein levels, and enhanced HBV cccDNA transcription. As reported before, HBx could recruit p300/CBP acetyltransferase to cccDNA, resulting in the acetylation of histone, consequently activating transcription (50). HBx-targeted degradation of the host restriction factor Smc5/6 promotes transcriptional activity of cccDNA (31). Hence, HBx may act as a promising antiviral target that, if inhibited, would repress HBV cccDNA transcription. In this study, we demonstrated that VCPIP1 recruiting PSMC3 promoted the HBV cccDNA transcription by inhibiting HBx degradation, which suggested that drugs or methods targeting VCPIP1 and PSMC3 may produce better efficacy to antagonize HBV cccDNA.

In summary, VCPIP1 is a novel DUB that stabilizes HBx in a ubiquitin-independent manner, serving as a scaffold for the interaction between free proteasome subunits PSMC3 and HBx. The pattern of binding of HBx by the 26S proteasome has been deciphered from the structural aspect for the first time and may provide new insights for developing UPS-based therapeutics for HBV-mediated pathogenesis.

## MATERIALS AND METHODS

### Plasmid construction.

pGEX-4T-1-HBx encoding the GST-HBx fusion protein, pRep-HBV comprising a 1.2-fold length of 3.2-kb HBV genome, pDsRed-Monomer-Hyg-N1-HBx encoding HBx fused with red fluorescent protein in the N terminus of the pDsRed-Monomer-Hyg-N1, pHA-Ub encoding ubiquitin with an N-terminal hemagglutinin (HA) tag, and the reporter plasmids pAP-1-luc, pNF-κB-luc, and pNF-SP-1-luc were constructed as previously reported (16). Seventy-four DUB genes encoding five DUB subfamilies in the pBob-DUBs-Flag (51) were used as the PCR-amplified template, and then each DUB fragment was cloned into pGADT7 vector (TaKaRa, USA) using the ExoIII-assisted ligase-free cloning method (52). Table 1 lists the GenBank accession numbers of the 74 DUBs.

pVCPIP1-Flag, pVCPIP1-Myc and pAcGFP1-Hyg-N1-VCPIP1 were constructed by inserting the *VCPIP1* gene (GenBank accession no. NM_025054.4) fused with a Flag tag or a Myc tag in the C terminus to pBob-Flag or pcDNA3.1/myc-his(-)A or fused with a green fluorescent protein in the N terminus of pAcGFP1-Hyg-N1, respectively. pCMVTNT-VCPIP1-Flag for the *in vitro* translation and pVCPIP1-HA were constructed by respectively inserting the *VCPIP1* gene fused with a Flag tag or an HA tag into the C terminus of the plasmid pCMV-TNT (Promega, USA). A series of fragments encoding VCPIP1 aa 1 to 743, aa 744 to 862, aa 863 to 1221, aa 863 to 982, aa 983 to 1101, and aa 1101 to 1221 (19) were generated by inserting PCR-amplified genes into pGADT7 vector. pVCPIP1#C-Flag encoding the C-terminal aa 863 to 1221 of VCPIP1 was cloned into the pBob-Flag plasmid.

pPSMC3-Myc was constructed by inserting the *PSMC3* gene (GenBank accession no. NM_002804.4), amplified by a reverse transcription-PCR (RT-PCR) kit (TaKaRa) using the total RNA of Huh7 cells, into pcDNA3.1/myc-his(-)A in frame with the C-terminal Myc tag. The full length and subclones of PSMC3 encoding aa 1 to 439, aa 1 to 213, aa 214 to 439, and aa 355 to 439 (25) were inserted into pGBKT7 or pGADT7 vector.

A series of HBx deletion mutants, i.e., HBxΔ1–25 (aa 1 to 25 deletion), HBxΔ26–50 (aa 26 to 50 deletion), HBxΔ51–80 (aa 51 to 80 deletion), HBxΔ81–120 (aa 81 to 120 deletion), and HBxΔ121–154 (aa 121 to 154 deletion), were cloned into pGBKT7 (16). pHBx-Flag and pHBx-Myc encoding the C-terminal Flag-tagged HBx and C-terminal Myc-tagged HBx proteins, respectively, were constructed in pcDNA3.1/myc.his(-)A backbone. pHBxKOR-Flag was generated by replacing lysines 91, 95, 113, 118, and 140 with arginine in pHBx-Flag.

To obtain the purified His-tagged HBx-Myc, VCPIP1-Flag, or PSMC3-Flag proteins in the E. coli expression system, the recombinant plasmids pHis-HBx-Myc, pHis-VCPIP1-Flag, and pHis-PSMC3-Flag were separately constructed by cloning the HBx-Myc, VCPIP1-Flag, and PSMC3-Flag fragments, respectively, into pRSFDuet-1 vector (Invitrogen, USA) in frame with an N-terminal 6×His tag. Table 2 lists the sequences of the primers and relevant restriction sites used for all of the plasmid constructions.

**TABLE 2:** Sequences of the primers used in plasmid construction

Plasmid	Primer^*a*^
pCMVTNT-VCPIP1-Flag	F: 5′-CCGCTCGAGGCCACCATGTCTCAGCCGCCGCCGCC-3′
	R: 5′-CTAGTCTAGATTACTTGTCGTCATCGTCTTTGTAGTCAGAGTGATCCATTGGCTCAG-3′
pVCPIP1-Myc	F: 5′-ATATCTGCAGAATTCGCCACCATGTCTCAGCCGCCGCC-3′
	R: 5′-ACCGAGCTCGGATCCAGAGTGATCCATTGGCTCAG-3′
pVCPIP1#C-Flag	F: 5′-ATCGGATCTGAATTCACTGTTTCTCCCAGTACCA-3′
	R: 5′-TCTGGCCAACTCGAGTTACTTGTCGTCATCGTCTTTGTAGTCAGAGTGATCCATTGGCTCAG-3′
pHis-HBx-Myc	F: 5′-CAGGGGCCCGAATTCATGGCTGCTAGGCTGTGCTG-3′
	R: 5′-TTTACCAGACTCGAGCAGATCCTCTTCTGAGATGAGTTTCTGCTCGGCAGAGGTGAAAAAGTTG-3′
pHis-PSMC3-Flag	F: 5′-CAGGGGCCCGAATTCATGAATCTGCTGCCGAATATTG-3′
	R: 5′-TTTACCAGACTCGAGTTACTTGTCGTCATCGTCTTTGTAGTCGGCGTAGTATTGTAGGTTG-3′
pHis-VCPIP1-Flag	F: 5′-CAGGGGCCCGAATTCATGTCTCAGCCGCCGCCGC-3′
	R: 5′-TTTACCAGACTCGAGTTACTTGTCGTCATCGTCTTTGTAGTCAGAGTGATCCATTGGCTCAG-3′
pGADT7-VCPIP1 (aa 1–1221)	F: 5′-AGTGAATTCCACCCGGTTAACTCTAGAGAATTCGGATCC-3′
	R: 5′-ATCGATGCCCACCCAAGCTTCCATGGCTCGAG-3′
pGADT7-VCPIP1 (aa 1–743)	F: 5′-GAGGCCAGTGAATTCTCTCAGCCGCCGCCGCCGC-3′
	R: 5′-GAGCTCGATGGATCCTCACCTGGGTTGCCCTTTTTGT-3′
pGADT7-VCPIP1 (aa 744–1221)	F: 5′-GAGGCCAGTGAATTCACTGTTTCTCCCAGTACCA-3′
	R: 5′-GAGCTCGATGGATCCTCAAGAGTGATCCATTGGC-3′
pGADT7-VCPIP1 (aa 744–862)	F: 5′-GAGGCCAGTGAATTCTCTCAGCCGCCGCCGCCGC-3′
	R: 5′-GAGCTCGATGGATCCTCACCTGGGTTGCCCTTTTTGT-3′
pGADT7-VCPIP1 (aa 863–1221)	F: 5′-GAGGCCAGTGAATTCACTGTTTCTCCCAGTACCA-3′
	R: 5′-GAGCTCGATGGATCCTCAAGAGTGATCCATTGGC-3′
pGADT7-VCPIP1 (aa 863–982)	F: 5′-GAGGCCAGTGAATTCCACTCAGCCCACACTGTGAAAC-3′
	R: 5′-GAGCTCGATGGATCCTTATACAGCCTCTTTAACTAAAT-3′
pGADT7-VCPIP1 (aa 983–1101)	F: 5′-GAGGCCAGTGAATTCAGTCAGGTTCGAGCAGAGG-3′
	R: 5′-GAGCTCGATGGATCCTTAAACCAAATCAGGATCAAGC-3′
pGADT7-VCPIP1 (aa 1102–1221)	F: 5′-GAGGCCAGTGAATTCGAGGCCCAGCGAAAAAAATTGC-3′
	R: 5′-GAGCTCGATGGATCCTTAAGAGTGATCCATTGGC-3′
pGADT7-PSMC3 (aa 1–439)	F: 5′-GAGGCCAGTGAATTCAATCTGCTGCCGAATATTG-3′
	R: 5′-CAGCTCGATGGATCCCTAGGCGTAGTATTGTAGG-3′
pGADT7-PSMC3 (aa 1–213)	F: 5′-GAGGCCAGTGAATTCAATCTGCTGCCGAATATTG-3′
	R: 5′-CAGCTCGATGGATCCTTACTCAAACTTCTCCTTG-3′
pGADT7-PSMC3 (aa 214–439)	F: 5′-GAGGCCAGTGAATTCAACTTGGGGATCCAACCTC-3′
	R: 5′-CAGCTCGATGGATCCCTAGGCGTAGTATTGTAGG-3′
pGADT7-PSMC3 (aa 355–439)	F: 5′-GAGGCCAGTGAATTCTTCCCGATGCCCAATGAGG-3′
	R: 5′-CAGCTCGATGGATCCCTAGGCGTAGTATTGTAGG-3′
pGBKT7-PSMC3 (aa 1–439)	F: 5′-ATGGAGGCCGAATTCAATCTGCTGCCGAATATTG-3′
	R: 5′-ATGCGGCCGCTGCAGCTAGGCGTAGTATTGTAGG-3′
pGBKT7-PSMC3 (aa 1–213)	F: 5′-ATGGAGGCCGAATTCAATCTGCTGCCGAATATTG-3′
	R: 5′-ATGCGGCCGCTGCAGTTACTCAAACTTCTCCTTG-3′
pGBKT7-PSMC3 (aa 214–439)	F: 5′-TGGAGGCCGAATTCAACTTGGGGATCCAACCTC-3′
	R: 5′-ATGCGGCCGCTGCAGCTAGGCGTAGTATTGTAGG-3′
pGBKT7-PSMC3 (aa 355–439)	F: 5′-ATGGAGGCCGAATTCTTCCCGATGCCCAATGAGG-3′
	R: 5′-ATGCGGCCGCTGCAGCTAGGCGTAGTATTGTAGG-3′
pCMVTNT-VCPIP1-HA	F: 5′-CGTGGTACCTCTAGAGCCACCATGTCTCAGCCGCCGCCGC-3′
	R: 5′-GCTCGAAGCGGCCGCTCAAGCGTAGTCTGGGACGTCGTATGGGTAAGAGTGATCCATTGGCTCAG-3′
GAPDH-qPCR	F: 5′-TGCACCACCAACTGCTTAGC-3′
	R: 5′-AGCTCAGGGATGACCTTGCC-3′
VCPIP1-qPCR	F: 5′-AACCATGGGTATGGCTGATG-3′
	R: 5′-AGTAGCCTCTGCTCGAACCT-3′
Total HBV RNA-qPCR	F: 5′-ACCGACCTTGAGGCATACTT-3′
	R: 5′-GCCTACAGCCTCCTAGTACA-3′
HBV pgRNA-qPCR	F: 5′-GCCTTAGAGTCTCCTGAGCA-3′
	R: 5′-GAGGGAGTTCTTCTTCTAGG-3′
HBV cccDNA-qPCR	F: 5′-GGGGCGCACCTCTCTTTA-3′
	R: 5′-CCACCCAGGTAGCTAGAGTCATTAG-3′

a Underlined sequences represent the restriction enzyme recognition sites of vectors.

### Yeast two-hybrid assay.

The yeast two-hybrid assay was performed according to the Matchmaker Gold yeast two-hybrid system user manual (TaKaRa). Briefly, to define DUBs that may interact with HBx, the Y2HGold strain transformed with pGBKT7-HBx was mated individually with Y187 containing pGADT7-DUBs. After mating for 22 h, the diploid cells were applied on plates with the selection medium QDO/AbA (SD/-Trp/-Leu/-His/-Ade containing 125 ng/mL aureobasidin A). Positive controls (pGADT7-T in Y187 strain mated with pGBKT7-53 in Y2HGold strain) and negative controls (pGADT7-T with pGBKT7-lam) were used.

### Cell culture and transfections.

Huh7 human hepatoma cells and HepG2 cells as well as HepG2.2.15, HepAD38, and HepG2-NTCP cells were respectively maintained in Dulbecco’s modified Eagle’s medium (DMEM) with 10% fetal bovine serum (FBS; PAN, Germany) and cultured in 5% CO_2_ at 37°C. Cells were transfected with the appropriate plasmids at a confluence of 80% using Lipofectamine 3000 reagent (Invitrogen).

### HBV production and infection.

HBV particles were harvested from the supernatant of HepAD38 cells as previously described (53). Briefly, supernatants were collected and combined with polyethylene glycol (PEG) 8000 (Sigma-Aldrich) at a final concentration of 8%, and then the mixture was rotated overnight at 4°C for 12 to 16 h and then centrifuged for 30 min at 4°C, 10,000 × *g*. The pellet containing HBV particles was redissolved in DMEM at 1% of the original sample volume. For infection, HepG2-NTCP cells were seeded in 6-well plates in DMEM supplemented with 10% FBS and 1 μg/mL doxycycline (TaKaRa). After 72 h, the cells were infected with HBV particles at a multiplicity of infection (MOI) of 200 mixed with 8% PEG solution containing 1.5% dimethyl sulfoxide (DMSO) (Sigma-Aldrich) to promote the HBV particle infection.

### GST pulldown assay.

GST and GST-tagged HBx proteins were obtained in the E. coli strain Rosetta(DE3) and then purified as described previously (24). Flag-tagged VCPIP1 proteins were transcribed and translated using 1 μg pCMVTNT-VCPIP1-Flag as the template by the TNT T7 Quick Coupled transcription-translation system (Promega, WI) following the manufacturer’s instructions. The translated VCPIP1 proteins were respectively mixed with GST-HBx or GST proteins immobilized in glutathione Sepharose 4B beads (GE Healthcare, Germany) as previously reported (24), and the interacting proteins were detected with anti-Flag antibody (CST, USA).

### Coimmunoprecipitation assay.

To validate the HBx-VCPIP1 interactions *in vivo*, Huh7 cells were harvested after 48 h of cotransfection with 3 μg pVCPIP1-Myc and 1.5 μg pHBx-Flag or empty vector pcDNA3.1/myc.his(-)A using Lipofectamine 3000. Correspondingly, 1.5 μg of pHBx-Myc and 3 μg of pVCPIP1-Flag or empty vector pBob-Flag were cotransfected into cells and then were lysed with Western and immunoprecipitation (IP) lysis buffer (Beyotime, China) containing phenylmethylsulfonyl fluoride (PMSF) protease inhibitor plus cocktail (Roche, Switzerland). The cleared 1-mg lysates obtained by centrifugation were immunoprecipitated using 20 μL EZview Red anti-Flag M2 affinity gel (Sigma, USA) as previously described (24). The immunoprecipitated complexes were eluted and analyzed with anti-Myc (CST) or anti-Flag antibodies by Western blotting.

### Confocal microscopy assay.

Huh7 cells cultured in glass-bottomed dishes (Thermo Scientific) were transfected with 0.5 μg pAcGFP1-Hyg-N1-VCPIP1 alone or cotransfected with 0.25 μg pDsRed-Monomer-Hyg-N1-HBx. After 42 h, cells were washed and incubated with 5 μM Hoechst 33342 (Thermo Scientific) in phosphate-buffered saline (PBS) at 37°C for 5 min and then fixed in methanol. Confocal microscopy was performed with a Zeiss LSM-410 laser scanning microscope.

### Western blotting.

Cells were harvested and lysed for 30 min at 4°C. The cell lysate supernatants were isolated by centrifugation. The same amounts of proteins were resolved by SDS-PAGE and transferred to polyvinylidene difluoride membranes (GE Healthcare). The membranes were incubated separately with the primary antibodies, followed by incubation with horseradish peroxidase (HRP)-coupled secondary antibody. The chemiluminescence of the bands was observed using the enhanced chemiluminescence (ECL) detection reagents (Amersham, UK). The band density was analyzed using ImageJ version 4.4.1 (National Institutes of Health, USA).

### Real-time qRT-PCR.

The total RNAs were extracted from Huh7 or HepG2 cells using TRIzol reagent and then reverse transcribed to cDNA with the ExScript RT-PCR kit (TaKaRa) according to the manufacturer’s instructions. The quantitative RT-PCR (qRT-PCR) was performed in duplicate with the SYBR Premix *Ex Taq* kit (TaKaRa). The relative mRNA levels were determined by the threshold cycle (2^−ΔΔ^*^CT^*) method (54). Table 2 shows the specific primers used for amplification.

### RNA interference.

siRNA duplexes for VCPIP1 and PSMC3 and control duplex siRNA were synthesized by Jima Biotechnology (Shanghai, China). The cells were transfected with 100 pmol siRNA for 4 × 10^5^ cells using Lipofectamine 3000. For *VCPIP1* knockdown, two targeted RNAi oligonucleotides, siVCPIP1#1 (5′-GCAUAAUACAGGGACAGACTT-3′ and 5′-GUCUGUCCCUGUAUUAUGCTT-3′) and siVCPIP1#2 (5′-GAAUGGAGAAUCUAGACAUTT-3′ and 5′-AUGUCUAGAUUCUCCAUUCTT-3′) were used. For *PSMC3* knockdown, siPSMC3 (5′-GCUGGUGCAGAUGUUCAUUTT-3′ and 5′-AAUGAACAUCUGCACCAGCTT-3′) was used. The nonsilencing RNAi oligonucleotides used as the negative control were termed siNC (5′-UCCUCCGAACGUGUCACGUTT-3′ and 5′-ACGUGACACGUUCGGAGAATT-3′), which encoded a sequence of no significant similarity to any known human, mouse, or rat genes.

### Determination of HBx half-life.

Huh7 or HepG2 cells were cotransfected with 0.75 μg pHBx-Myc or 0.75 μg pHBxKOR-Flag and 1.5 μg pVCPIP1-Flag or 1.5 μg pVCPIP1#C-Flag or 100 pmol siVCPIP1#1. At 36 h posttransfection, 100 μg/mL cycloheximide (Sigma) was incubated with the cells for 0, 30, 45, 60, 90, or 120 min. The HBx protein levels were detected by Western blotting.

### Ubiquitylation assay.

Huh7 and HepG2 cells were cotransfected with 1 μg pHA-Ub, 0.75 μg pHBx-Flag, and 1.5 μg pVCPIP1-Myc or empty vector. After 42 h, the cells were treated with MG132 (Sigma) at a concentration of 20 μM for another 6 h. The polyubiquitination level of HBx was detected by the immunoprecipitation method using EZview Red anti-Flag M2 affinity gel and analyzed with anti-ubiquitin antibody, anti-K48-linked ubiquitin antibody, and anti-K63-linked ubiquitin antibody (CST) as described previously (24). Additionally, the cells were cotransfected with 1 μg pHA-Ub, and 0.75 μg pHBxKOR-Flag or pHBx-Flag, and treated with MG132, and then the extracted proteins were immunoprecipitated and detected with anti-HA antibody to evaluate the HBxKOR polyubiquitination level.

### Nickel affinity purification.

The recombinant plasmid pHis-HBx-Myc, pHis-PSMC3-Flag, or pHis-VCPIP1-Flag was transformed into the competent E. coli DE3 cells. The purified N-terminal hexahistidine (His)-tagged HBx-Myc or PSMC3-Flag or VCPIP1-Flag proteins were obtained by nickel affinity purification using chelating Sepharose Fast Flow beads (GE Healthcare) following the instructions of the manufacturer.

### *In vitro* HBx protein degradation assay.

To test His-tagged HBx degradation *in vitro*, 10 μg purified proteins was treated with or without 1 μg 20S proteasome (LifeSensors, USA) for 3 h or 5 h at 37°C as previously described (46). A tube of mixed proteins was additionally supplemented with 10 μM MG132. Similarly, 10 μg purified His-HBx-Myc proteins and 10 μg purified His-PSMC3-Flag proteins or 20 μg His-VCPIP1-Flag proteins were mixed on ice for 10 min to establish protein association *in vitro*, and then 1 μg 20S proteasome was added and incubated for 3 h at 37°C. Subsequently, the HBx protein levels were detected with anti-Myc antibody.

### *cis*-Element luciferase reporter assay.

Huh7 and HepG2 cells were cotransfected with 0.05 μg pRL-TK and 0.5 μg pNF-κB-luc, or pAP-1-luc, or pSP-1-luc luciferase reporter plasmids together with 0.25 μg pHBx-Myc or 0.5 μg pVCPIP1-Flag for 48 h. Firefly and *Renilla* luciferase activities were measured using a dual-luciferase reporter assay system (Promega). For each sample, the firefly luciferase activity was calibrated to that of *Renilla* luciferase and then standardized to the empty vector.

### Colony formation assay.

Huh7 and HepG2 cells were transfected with 0.375 μg pHBx-Flag or 0.75 μg pVCPIP1-Myc or both. After 48 h, the cells were selected in medium containing G418 for 21 days. The cells were then stained with crystal violet staining solution (Beyotime, China). G418-resistant colonies were counted and photographed.

### HBV cccDNA extraction and quantification.

The Hirt DNA extraction methods were employed for cccDNA extraction from the HBV particle-infected HepG2-NTCP cells (55). Then, the extracts were incubated with plasmid-safe ATP-dependent DNase (PSAD) (Lucigen, USA) to hydrolyze linear double-stranded DNA. Subsequently, cccDNA in the PSAD-treated samples was analyzed by real-time quantitative PCR assay with specific primers listed in Table 2.

### HBV DNA quantification and ELISA detection.

The HBV DNA quantification in the culture supernatant of HBV-infected HepG2-NTCP cells was determined according to the instructions with the hepatitis B viral DNA quantitative fluorescence diagnostic kit (Santurebiotech, China). The expression levels of HBsAg and HBeAg in the culture supernatant of HBV-infected HepG2-NTCP cells were determined with human HBsAg and HBeAg enzyme-linked immunosorbent assay (ELISA) kits (Andy Gene, China) following the manufacturer’s instructions.

### Statistical analysis.

SPSS 19.0 software (SPSS Inc., USA) was used for all statistical analyses. Student’s *t* test was used to analyze differences between two groups. Statistical analysis of pairwise comparisons between groups was performed by a one-way analysis of variance (ANOVA). All values in the graph are expressed as the means ± standard deviations (SD) from triplicate experiments. A *P* value of <0.05 was considered statistically significant. Experiments were performed at least three times.
